# Performance of machine learning software to classify breast lesions using BI-RADS radiomic features on ultrasound images

**DOI:** 10.1186/s41747-019-0112-7

**Published:** 2019-08-05

**Authors:** Eduardo Fleury, Karem Marcomini

**Affiliations:** 1 Instituto Brasileiro de Controle do Câncer (IBCC), São Paulo, Brazil; 2 Centro Universitário São Camilo, Curso de Medicina, São Paulo, Brazil; 3 USP São Carlos, São Paulo, Brazil

**Keywords:** Breast neoplasms, Machine Learning, Neural networks (computer), Support vector machine, Ultrasonography

## Abstract

**Background:**

The purpose of this work was to evaluate computable Breast Imaging Reporting and Data System (BI-RADS) radiomic features to classify breast masses on ultrasound B-mode images.

**Methods:**

The database consisted of 206 consecutive lesions (144 benign and 62 malignant) proved by percutaneous biopsy in a prospective study approved by the local ethical committee. A radiologist manually delineated the contour of the lesions on greyscale images. We extracted the main ten radiomic features based on the BI-RADS lexicon and classified the lesions as benign or malignant using a bottom-up approach for five machine learning (ML) methods: multilayer perceptron (MLP), decision tree (DT), linear discriminant analysis (LDA), random forest (RF), and support vector machine (SVM). We performed a 10-fold cross validation for training and testing of all classifiers. Receiver operating characteristic (ROC) analysis was used for providing the area under the curve with 95% confidence intervals (CI).

**Results:**

The classifier with the highest AUC at ROC analysis was SVM (AUC = 0.840, 95% CI 0.6667–0.9762), with 71.4% sensitivity (95% CI 0.6479–0.8616) and 76.9% specificity (95% CI 0.6148–0.8228). The best AUC for each method was 0.744 (95% CI 0.677–0.774) for DT, 0.818 (95% CI 0.6667–0.9444) for LDA, 0.811 (95% CI 0.710–0.892) for RF, and 0.806 (95% CI 0.677–0.839) for MLP.

Lesion margin and orientation were the optimal features for all the machine learning methods.

**Conclusions:**

ML can aid the distinction between benign and malignant breast lesion on ultrasound images using quantified BI-RADS descriptors. SVM provided the highest ROC-AUC (0.840).

## Key points

- Five different machine learning classifiers were utilised to differentiate malignant from benign breast lesions on B-mode ultrasound images using ten BI-RADS features. The area under the curve obtained by machine learning systems ranged from 0.806 to 0.840.
- The best performance was obtained by the support vector machine system with an area under the curve of 0.840, 71.4% of sensitivity, and 76.9% of specificity.
- Machine learning systems based on BI-RADS feature can help in malignant/benign differentiation but further improvement is needed.

## Background

Ultrasound imaging is one of the most effective tools as an adjunct to mammography to detect and diagnose breast abnormalities. It is useful to detect and distinguish benign from malignant masses with high accuracy, reducing the number of unnecessary biopsies [1, 2].

Since 2003, the American College of Radiology developed the Breast Imaging and Reporting Data System (BI-RADS) ultrasound lexicon that provides standard terminology to describe the findings in relation with the probability of malignancy [3, 4]. The dominant sonographic characteristics are described according to five BI-RADS descriptive categories: shape, orientation, margins, echo pattern, and posterior acoustic transmission [5, 6].

One of the aims of radiomics is to extract, process, and classify a number of imaging features in order to determine the phenotypic characteristics of a lesion that helps to differentiate malignant from benign lesions. Radiomics can be used for any imaging method, including ultrasound scan [7].

Advances in the field of image processing have aided to improve sensitivity and specificity [8]. Several software have been developed to quantify lesion characteristics [9–11] related to shape and texture. Other studies have tried to quantify the features used by the radiologists by “translating” the descriptive terms from the BI-RADS lexicon into computerised features so that the algorithms can automatically compute these features [1, 6, 8]. The authors consider that the main advantage given by these systems using BI-RADS sonographic characteristics is that the system could be applied on images provided by different ultrasound equipment [6]. In this context, machine learning can be broadly defined as computational methods/models using experience (data) to improve performance or make accurate predictions.

The purpose of this work was to assess whether BI-RADS computerised features can improve the diagnosis by computational decision, using five different machine learning methods.

## Methods

This prospective study was approved by the Research Ethics Committee of Brazilian Institute for Cancer Control (IBCC—São Paulo, SP, Brazil) (protocol number 012664/2016) and was registered in the Plataforma Brazil (protocol number 53543016.2.0000.0072). We obtained informed consent from all included patients and protected their private information.

The cases were prospectively collected from September 2017 to July 2018 during diagnostic breast exams at the IBCC. The population consisted of 144 women (43.6 ± 11.1 years, mean ± standard deviation) with 206 solid lesions, 144 being benign and 62 being malignant at percutaneous core biopsy. The histopathology results of the benign and benign lesions are listed in Table 1. We used four ultrasound systems to acquire the images: Toshiba Nemio 30, Toshiba Aplio 400 (Toshiba, Tokyo, Japan), Siemens VFX 13-5, and Siemens FV 10-5 (Siemens, Erlangen, Germany), with 5-10 MHz linear transducers. A radiologist with 2 years of experience in breast imaging performed the ultrasound exams.

**Table 1:** Histopathology of the 206 solid lesions at percutaneous core-biopsy

Bening lesions			Malignant lesions			
Type	Number	Percentage (%)	Type		Number	Percentage (%)
Fibroadenoma	71	49.3	Ductal carcinoma *in situ*		1	1.6
Fibocystic changes	48	33.3	Invasive ductal carcinoma	GI	9	14.5
Phyllodes tumour	3	2.1		GII	33	53.2
Papylary lesion	3	2.1		GIII	14	22.6
Other^$^	19	13.2	Invasive lobular carcinoma		5	8.1
Total	144	100.0	Total		62	100.0

^**$**^Steatonecrosis, mastitis, fat tissue

### Feature extraction and selection

Five main sonographic mass features are described in the BI-RADS lexicon fifth edition: shape, orientation, margin, echo pattern, and posterior acoustic features [12].

We used ten BI-RADS computerised features that were proposed by Shan et al. [8]: {1} area of difference with equivalent ellipse (ADEE), {2} lesion orientation, {3} average of difference vector (AvgDiff), {4} number of peaks on the distance vector (NumPeaks), {5} average of the distance vector (AvgDistance), {6} area difference between the convex hull and tumour (ADCH), {7} echogenicity, {8} entropy, {9} shadow, and {10} lesion size. These multiple computerised features are proposed as discussed below.

**Fig. 1:**
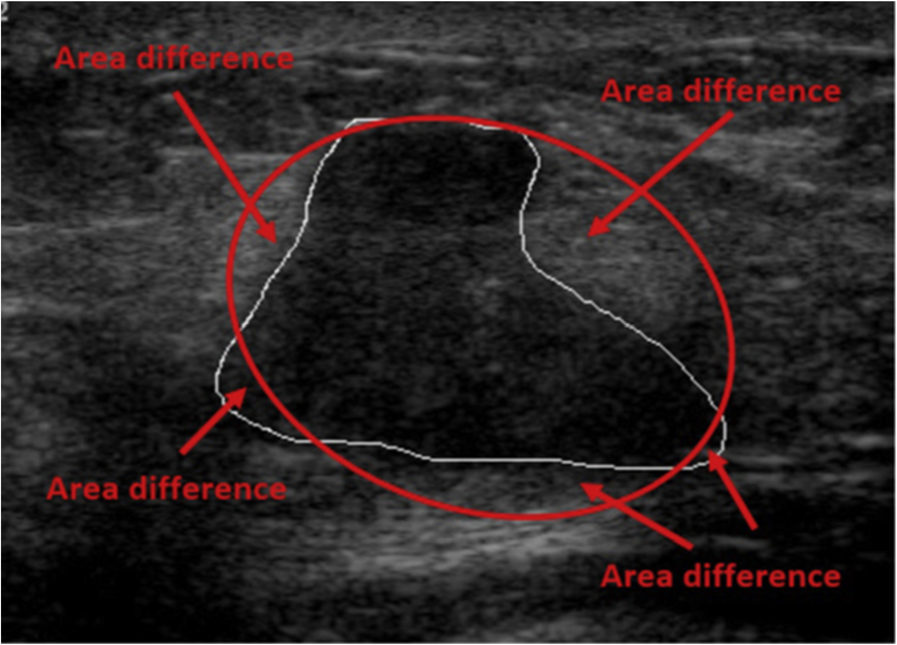
Area difference between a breast malignant mass and its equivalent ellipse

According to the BI-RADS lexicon, the breast mass shape can be round, oval, or irregular. Irregular shape is a sign suggestive of malignancy. We used an equivalent ellipse with the same second moments as the mass area and calculated the ADEE, defined as:

$$ \mathrm{ADEE}=\frac{A_E+{A}^T-{A}_{E\cap T}}{A^T} $$

where *A*_*E*_ is the number of pixels in the equivalent ellipse, *A*^*T*^ the number of pixels in the tumour region, and *A*_*E* ∩ *T*_ the number of pixels in the intersection between the tumour and the ellipse. Figure 1 illustrates the area difference between the tumour and its equivalent ellipse: the more irregular the shape, the greater the area of difference.

Orientation identifies the direction of the longer lesion axis. It can be perpendicular to the skin layer, *i.e*., the lesion is taller than wide (a sign of malignancy), or parallel to the skin layer, *i.e*., the lesion is wider than tall (a sign of benignancy). To quantify this feature, we used the following equation:

$$ \mathrm{Orientation}=\frac{\mathrm{Height}}{\mathrm{Width}} $$

**Fig. 2:**
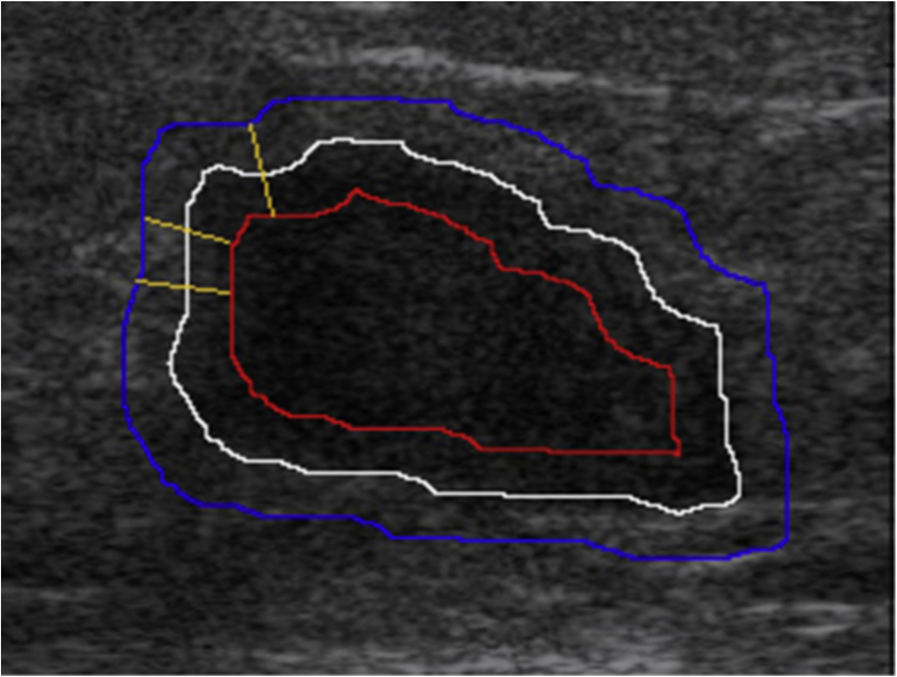
Original contour (white), inside contour (red), and outline contour (blue) of a lesion

Margin characteristics are an excellent BI-RADS descriptor predictor of malignancy, including several subcategories as follows: indistinct, angular, microlobulated, and spiculated. Indistinct margin is related to no clear demarcation between a mass and its surrounding tissue. To compute this feature, we defined the intensity difference vector drawing the outside and inside contour along the tumour contour with a 20-pixel width on each side (Fig. 2, where the yellow lines represent three segments on which the intensity difference vector is computed; each segment starts from a pixel on the outside contour and ends up at the closest pixel on the inside contour). The intensity difference vector (Diff) is calculated as follows:

$$ \mathrm{Diff}(i)=\overline{I_{\mathrm{out}}(i)}-\overline{I_{\mathrm{in}}(j)} $$

where *i* is the *i*th pixel on the outside blue contour and *j* is the closest pixel to *i* on the inside red contour, $\overline{I_{\mathrm{out}}(i)}$ is the average intensity of pixels on the outside half of the line segment *ij*, and $\overline{I_{\mathrm{in}}(j)}$ is the average intensity of pixels on the other half of the line segment *ij*) (see Fig. 2).

The computerised indistinct margin feature can be represented by the average of vector Diff, that is:

$$ \mathrm{AvgDiff}=\frac{\sum_{i\in \mathrm{out}}\mathrm{Diff}(i)}{N} $$

where *i* is a pixel on the outside contour, and *N* is the number of pixels on the outside contour.

The other margin related features (angular, microlobulated, or spiculated) are related to the contour smoothness. The common characteristic of these irregular shapes is captured by a proposed digital feature. The distance vector between the tumour contour and its convex hull is computed by a drawn of the convex hull of the tumour. The distance to the closest point on the tumour contour is saved in the distance vector *V*convex:

$$ {V}_{\mathrm{convex}}(i)=\sqrt{{\left({x}_i-{x}_j\right)}^2+{\left({y}_i-{y}_j\right)}^2} $$

where *i* is the *i*th pixel on the convex hull, *j* is the closest pixel to *i* on the tumour contour, and *x* and *y* are the coordinates of the pixels.

**Fig. 3:**
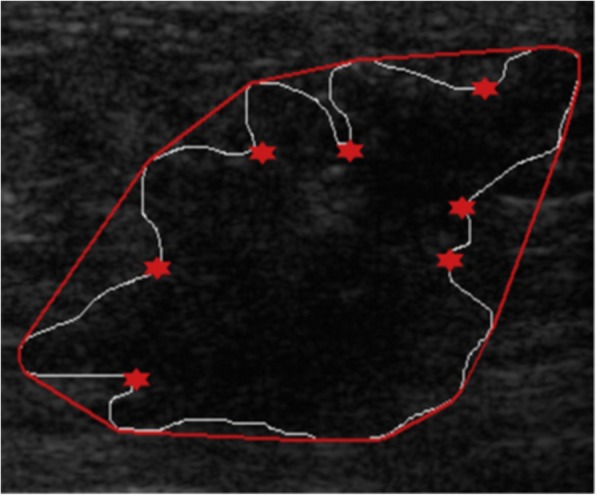
Convex hull (red contour) of a malignant lesion (white contour), with peaks on the distance vector. *V*convex are marked by stars

We extracted three features from the distance vector to describe the margin: the number of peaks (NumPeaks), the average of the distance vector (AvgDistance), and the area difference between the convex hull tumour (ADCH). A higher NumPeaks means that the contour is bumpier; a higher AvgDistance indicates a spiculated contour; a higher ADCH indicates irregularity in the contour. Figure 3 shows how the number of peaks on the distance vector corresponds to the number of valleys on the tumour contour, which are marked by red stars. These three digital features are defined as follows:

$$ \mathrm{NumPeaks}=\mathrm{Number}\ \mathrm{of}\ \mathrm{local}\ \mathrm{maxima}\ \mathrm{of}\ {V}_{\mathrm{convex}} $$

$$ \mathrm{AvgDistance}=\mathrm{Average}\ \mathrm{of}\ {V}_{\mathrm{convex}} $$

$$ \mathrm{ADCH}=\frac{A_c-{A}_T}{A_T} $$

where *V*_convex_ is the distance vector between the tumour boundary and the corresponding convex hull, *A*_*c*_ is the number of pixels within the convex hull, and *A*_*T*_ is the number of pixels within the tumour (see Fig. 3).

Echo pattern, the average intensity of the tumour and the surrounding tissues provides a reference to describe the degree of echogenicity and might be captured by the following index:

$$ \mathrm{Echogenicity}={\mathrm{AvgIntensity}}_{\mathrm{surrounding}}-{\mathrm{AvgIntensity}}_{\mathrm{tumour}} $$

The surrounding region should be a rectangular region that contains the tumour in its centre and is about twice the size of the tumour. Shadow areas should be excluded from the surrounding region to provide an accurate reference. A positive echogenicity indicates that the tumour is hyperechoic whereas a negative echogenicity indicates that the tumour is hypoechoic.

The heterogeneous ultrasound pattern is a combination of darker and lighter components. The information obtained from the entropy refers to the probability distribution of grey values. A low entropy value corresponds to an image with a few information, *i.e*., has low variability of intensities values (prevalent homogeneity), while a high entropy value corresponds to an image containing a lot of information, *i.e*., different intensities values (prevalent heterogeneity). The entropy feature is proposed to describe the degree of heterogeneity:

$$ \mathrm{Entropy}=-\sum \limits_i{P}_i{\log}_2{P}_i $$

where *P*_*i*_ is the probability that the intensity difference between two adjacent pixels is equal to *i*.

Acoustic shadowing is considered worrisome for malignancy. For measuring the posterior acoustic feature, we determined a rectangular region below the tumour (with a size similar to that of the tumour) and compared its average intensity with that of the tumour. If the difference is positive, it means no shadow, whereas a negative difference indicates the presence of shadow.

$$ \mathrm{Shadow}=\overline{I_{\mathrm{post}}}-\overline{I_{\mathrm{tumour}}} $$

where $\overline{I_{\mathrm{post}}}$ is the average intensity level of the rectangular region below the tumour and with similar size to the tumour.

Lesion size is not a standard BI-RADS feature [12]. However, for automatic tumour diagnosis, lesion size can improve the performance of lesions classifiers when combined with other features. We represented it by the number of pixels within the tumour contour.

**Fig. 4:**
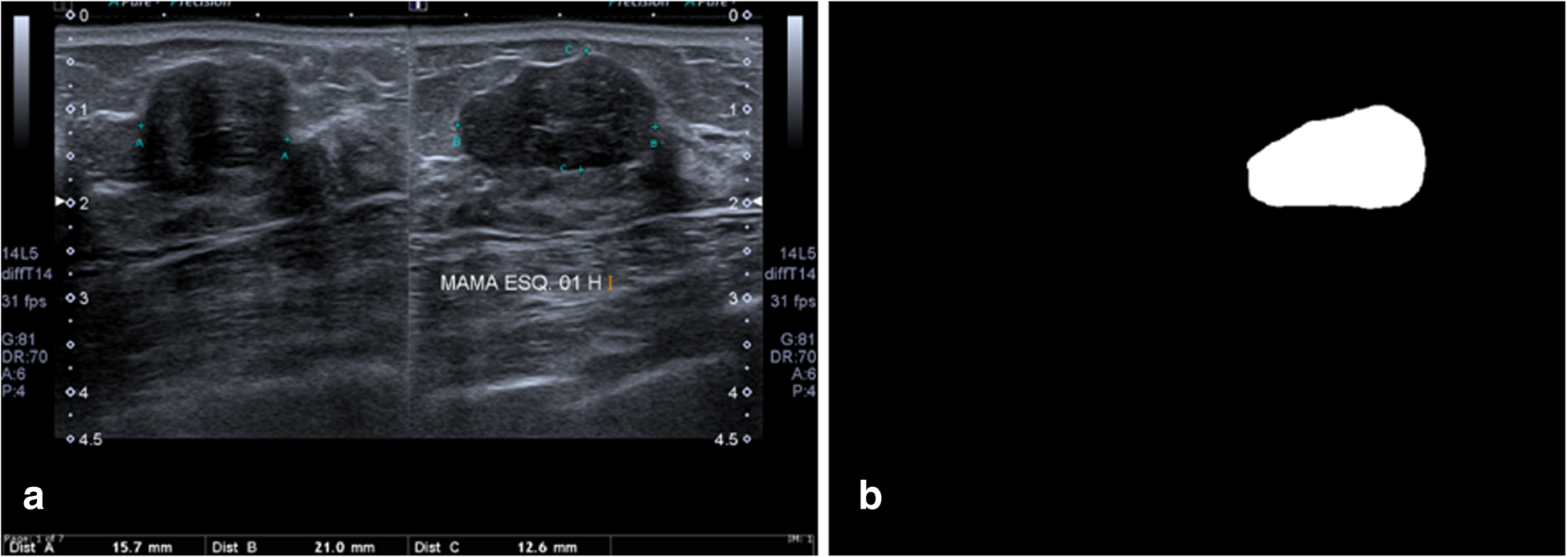
Example of segmentation of a fibroadenoma. Original (**a**) and segmented (**b**) images (to classify the lesions, the segmented image was along the plane best representing the mass that was used, in this case, the long axis)

Thus, we calculated ten features related to morphology and texture tumour based on the BI-RADS lexicon. The features were calculated starting from the lesion segmentation performed by a single operator with 16 years of experience in breast imaging (Fig. 4).

### Lesions classification

In this study, we applied five machine learning methods to distinguish between benign and malignant lesions using the previously described features.

Decision tree (DT) [13] is a decision support tool that uses a tree-like graph and its possible consequences. It is a rule-based decision model. This algorithm was implemented in the Weka Package [14]. Random forest (RF) [15] operates by constructing a multitude of decision trees during the training phase and outputting the class that is the overall prediction of the individual trees. This method can correct the overfitting problem of decision trees. We used the algorithm included in the WEKA Package [14]. Artificial neural network (ANN) [16] is a self-learning method based on examples. It simulates the nervous system properties and biological learning functions through an adaptive process. It is composed of an input layer, one or more intermediate (or hidden) layers, and an output layer [17]. We used the model multilayer perceptron (MLP), included in the WEKA Package [14] with a backpropagation algorithm to update the weights. Linear discriminant analysis (LDA) [18] is used in pattern recognition tasks to find a linear combination to characterise or separate two or more classes of objects. It is also related to the analysis of variance. It has continuous independent variables and a dependent categorical variable. To implement this method, we used the “fitcdiscr” function included in MATLAB R20014a (MathWorks, Natick, USA). Support vector machine (SVM) [19] is a classification technique that attempts to find an ideal hyperplane to separate two classes in a sample. To train this method, we used the “fitcsvm” function included in MATLAB R20014a (MathWorks, Natick, USA).

We performed 10-fold cross validation for training and testing of all classifiers.

### Statistical analysis

We evaluated the different combinations of input features for each machine learning approach in order to select the one with the best classification performance. For this task, each feature was individually evaluated. We selected the one with the best classification performance, *i.e*., with the highest value of the area under the curve (AUC) at the receiver operating characteristic (ROC) analysis. Then, a new feature was incrementally added to the one previously selected and the algorithm was trained again with the new combination. We selected the combination with the highest AUC. The incremental addition of features occurred until there was a reduction in the classifying performance or until all features were already included. The ROC analysis was performed and 95% confidence intervals for AUCs were obtained using Med Calc software v16.2 (MedCalc Software, Ostend, Belgium). Sensitivity and specificity were calculated at the best cutoff.

## Results

Tables 2, 3, 4, 5 and 6 show the feature selection procedure for each machine learning method. To evaluate the best input vector for each classifier, we measured the values of sensitivity, specificity, and AUC.

**Table 2:** Performance of different feature combinations using the decision tree method

Features	Sensitivity (%)	Specificity (%)	Area under the curve
{6}	73.2	69.1	0.652
{6, 1}	71.2	69.2	0.653
{6, 1, 2}	71.9	73.3	0.720
{6, 1, 2, 8}	70.6	75.0	0.744

{1}: area difference with equivalent ellipse; {2}: orientation; {6}: area difference between the convex hull and tumour; {8}: entropy

**Table 3:** Performance of different feature combinations using the multilayer perceptron method

Features	Sensitivity (%)	Specificity (%)	Area under the curve
{5}	67.5	78.7	0.759
{5, 2}	68.4	79.2	0.789
{5, 2, 1}	68.8	84.1	0.799
{5, 2, 1, 3}	66.2	71.7	0.806

{1}: area difference with equivalent ellipse; {2}: orientation; {3}: average of difference vector; {5}: average of distance vector

**Table 4:** Performance of different feature combinations using the random forest method

Features	Sensitivity (%)	Specificity (%)	Area under the curve
{4}	62.2	71.7	0.697
{4, 8}	72.3	74.6	0.760
{4, 8, 5}	72.6	72.6	0.778
{4, 8, 5, 2}	72.7	75.9	0.811

{2}: orientation; {4}: number of peaks on the distance vector (NumPeaks); {5}: average of distance vector; {8}: entropy

**Table 5:** Performance of different feature combinations using the linear discriminant analysis method

Features	Sensitivity (%)	Specificity (%)	Area under the curve
{5}	59.5	87.4	0.770
{5, 2}	76.0	69.8	0.818

{2}: orientation; {5}: average of distance vector

**Table 6:** Performance of different feature combinations using the support vector machine method

Features	Sensitivity (%)	Specificity (%)	Area under the curve
{4}	64.3	80.5	0.746
{4, 2}	67.1	76.2	0.798
{4, 2, 10}	67.1	78.8	0.807
{4, 2, 10 ,8}	68.6	76.2	0.814
{4, 2, 10 ,8 ,1}	71.4	76.9	0.840

{1}: difference area with equivalent ellipse; {2}: orientation; {4}: number of peaks on the distance vector; {8}: entropy; {10}: lesion size

## Discussion

Machine learning systems are increasingly proposed for aiding imaging diagnosis. Studies showed that the double reading improves the diagnostic performance of breast lesions [20–22]. However, the operational cost of double reading performed by two radiologists practically precludes its application outside the organised screening mammography programs. Thus, if the second reading would be performed by a computational method, we could improve the performance of the examiner at a lower cost.

In the past, software performing computer-aided diagnosis provided unsatisfactory results. Computers were trained to classify the lesions as humans do. It was like trying to teach the computer to think like a human being. However, while the computer only makes processing objective data, the human brain utilises abstract senses related to vision as well as smell, touch, taste, and hearing. With the use of machine learning systems, it is now possible to make an analogy of the subjective data used by humans with objective information used by computers. In this way, the computer can classify the lesions in an analogous way to the human beings.

The entire process basically consists of 4 steps: (1) image acquisition, (2) data extraction, (3) data processing, and (4) classification.

In the current study, we tried to adapt information obtained through data extraction with the classifications proposed by the BI-RADS lexicon. We assumed that different learning methods could have different optimal sets of features. The experimental results confirmed this hypothesis.

Some features have low differentiation performance when used separately. However, they can improve the classifier performance when associated with other features. The entropy can be given as an example for this situation, because when it was used individually by the RF classifier, it yielded the lowest AUC (0.481). On the other hand, when it was associated with other features, we observed an improvement in performance, as shown in Table 4.

Including some features in the input vector may make the classifier more sensitive or more specific, as in the case of the DT. When the feature orientation was added to the input vector, the classifier became more specific than sensitive (see Table 7).

**Table 7:** Performance of five different machine learning methods for classifying 206 solid breast lesion on ultrasound images

Method	Features	Sensitivity		Specificity		AUC	
		Point estimate (%)	95% CI	Point estimate (%)	95% CI	Point estimate	95% CI
Decision tree	{6, 1, 2, 8}	70.6	0.5889–0.8008	75.0	0.6231–0.8448	0.744	0.677–0.774
Multilayer perceptron	{5, 2, 1, 3}	66.2	0.5462–0.7612	71.7	0.5843–0.8203	0.806	0.677–0.839
Random forest	{4, 8, 5, 2}	72.7	0.5983–0.8181	75.9	0.593–0.811	0.811	0.710–0.892
Linear discriminant analysis	{5, 2}	76.0	0.6212–0.8345	69.8	0.6156–0.8316	0.818	0.6667–0.9444
Support vector machine	{4, 2, 10, 8, 1}	71.4	0.6479–0.8616	76.9	0.6148–0.8228	0.840	0.6667–0.9762

{1}: area difference with equivalent ellipse; {2}: orientation; {3}: average of difference vector; {4}: number of peaks on the distance vector; {5}: average of distance vector; {6}: area difference between the convex hull and tumour; {7}: echogenicity; {8}: entropy; {9}: shadow; {10}: lesion size. *CI* Confidence interval

The overall analysis has shown that proposed features have a higher ability to distinguish between benign and malignant lesions, especially orientation, NumPeaks, and AvgDistance, related to orientation and margin. Other features presented good potential when they were associated with the first ones. They are ADEE and entropy, related to the shape and ultrasound pattern.

We compared the results of the current study with those obtained by Shan et al. [8]. These authors tested ten BI-RADS features with the same classifiers used by us, except LDA. Their results are shown in Table 8. We can observe a variation of the best optimal set of features between both studies. With RF, for example, our optimal set of features was entirely different compared to the one selected by Shan et al. [8]. On the other hand, our optimal set for our ANN/MLP had four of the six features selected by Shan et al. [8]. These inter-study variations may be related to the way each specialist manually delineated the contour or with the image acquisition procedure, since the operator and the equipment were different.

**Table 8:** Performance of different machine learning methods obtained by Shan et al. [8]

Method	Features	Sensitivity (%)	Specificity (%)	Area under the curve
Decision tree	{4, 3, 10, 2, 7}	74.0	82.0	0.803
Multilayer perceptron	{4, 3, 2, 6, 5, 1}	78.0	78.2	0.823
Random forest	{6, 10, 2, 9, 3}	75.3	82.0	0.828
Support vector machine	{4, 2, 6, 3, 10}	77.3	78.2	0.842

The SVM was the machine learning method reaching the best performance in both studies, providing an AUC very close to each other (about 0.84). Other classifiers showed a slightly larger difference in the AUC. Therefore, we can consider that there was no negative influence on the use of images from different equipment to perform the training of machine learning methods, since the results are close to those presented in the current literature [23–25]. It is important to highlight that the performance by radiologists adopting descriptors defined by the fifth edition of BI-RADS to classify breast masses was reported in 2016 to be only 0.690 (ROC-AUC) [26].

Smart Detect is a commercial system that was recently developed by Samsung Medison (Seoul, Korea). This system provides assistance in the morphological analysis of breast masses seen on breast US according to BI-RADS descriptors. There are a few studies [23, 24, 27] that evaluate the diagnostic performance or the degree of agreement of Smart Detect with breast radiologists. In the study by Cho et al. [27], using the Smart Detect system, the authors achieved a sensitivity of 72.2%, a specificity of 90.8%, and an AUC of 0.815, a value slightly (-0.035, 3.5%) lower than the AUC obtained in the current study (AUC 0.840). We believe that the main reasons for this difference, although small, were as follows: (1) in the current study, we used 4 different equipment from 2 manufacturers and (2) the interpolation of benign lesions classified as malignant. This may have been the reasons that determined the difference in the mass classification criteria adopted in this study compared to Smart Detect (4, 2, 10, 8, 1 *versus* 4, 3, 2, 6, 5, 1). The present model recognised the lesion morphology and margins as the main classifier features. Because our model was calibrated using images of different equipment, we believe that would be more replicable in clinical practice. The features related to the margin showed a strong potential for the distinction between benign and malignant lesions using machine learning methods on ultrasound images, since its relevance was high for all the five methods discussed.

As a limitation of the present study, we mention the limited sample size, the way of the selection and combination of the features, and the use of 10-fold cross-validation as a single method to evaluate the model performance. As a future work, we intend to increase the number of samples from our image database to allow the use of other validation methods and ensure greater data reliability, especially by using an external dataset. Another perspective is to include new methods for selecting the best feature set. Finally, we intend to verify the classification performance through convolutional neural networks, eliminating the need for feature extraction and selection.

In conclusion, we showed machine learning algorithms applied to BI-RADS descriptors for ultrasound images of solid masses after lesion contouring by a breast radiologist which allow for differentiating malignant from benign tumours, with the SVM approach providing an AUC of 0.840.

## Abbreviations

ADCH: Area difference between the convex hull and tumour
ADEE: Area difference with equivalent ellipse
ANN: Artificial neural network
AUC: Area under the curve
AvgDiff: Average of difference vector
AvgDistance: Average of distance vector
BI-RADS: Breast Imaging and Reporting Data System
LDA: Linear discriminant analysis
MLP: Multilayer perceptron
NumPeaks: Number of peaks of distance vector
RF: Random forest
ROC: Receiver operating characteristic
SVM: Support vector machine
